# Pharmacokinetic Studies of Three Alkaloids in Rats After Intragastrical Administration of *Lycopodii Herba* Extract by LC-MS/MS

**DOI:** 10.3390/molecules24101930

**Published:** 2019-05-19

**Authors:** Dongke Ma, Xiaoting Gu, Xin Wang, Youping Liu, Xin Di

**Affiliations:** Laboratory of Drug Metabolism and Pharmacokinetics, Shenyang Pharmaceutical University, 103 Wenhua Road, Shenyang 110016, China; madongke225@163.com (D.M.); guxiaoting320@126.com (X.G.); wangxin68k@163.com (X.W.); yp-liu@163.com (Y.L.)

**Keywords:** *Lycopodii Herba*, lycodoline, α-obscurine, *N*-demethyl-α-obscurine, pharmacokinetics, brain tissue distribution, liquid chromatography–tandem mass spectrometry

## Abstract

*Lycopodii Herba* is a widely used traditional medicinal herb, and contains diverse fascinating alkaloids. In this study, a fast and sensitive LC-MS/MS method for the simultaneous determination of lycodoline, α-obscurine, and *N*-demethyl-α-obscurine from *Lycopodii Herba* in rat plasma and brain tissue was developed and validated. Biological samples were extracted via a protein precipitation procedure using methanol as the extraction solvent and Huperzine B as the internal standard. Chromatographic separation was carried out using a Thermo Syncronis-C18 column (50 mm × 2.1 mm, 5 μm) and a gradient mobile phase containing methanol and water with 0.05% formic acid. The three alkaloids were detected by positive electrospray ionization in selective reaction monitoring mode. The selectivity, crosstalk, carryover effect, linearity, accuracy, precision, extraction recovery, matrix effect, and stability of the current method were validated. Then, using the validated method, the plasma pharmacokinetics and brain tissue distribution of the alkaloids in rats were investigated after intragastrical administration of *Lycopodii Herba* extract. The three alkaloids were shown to be rapidly absorbed into the blood (*T*_max_, 0.79–1.58 h), and then also eliminated rapidly (*t*_1/2_, 1.27–2.24 h). All of them could pass through the blood–brain barrier. The method provides a new research approach to expand preclinical studies of *Lycopodii Herba*.

## 1. Introduction

*Lycopodii Herba* (the dried whole plant of *Lycopodium japonicum* Thunb., Lycopodiaceae) is a traditional medicinal herb that is found throughout eastern Asia, especially in the southern and eastern areas of China [1]. It has been officially recorded in the Chinese Pharmacopoeia; possesses anti-inflammation activity [2,3], antioxidative activity [4], and antitumor activity [5]; and has been traditionally used for the prevention and treatment of various diseases such as rheumatoid arthritis, contusion, quadriplegia, dysmenorrhea, and other health problems [6,7,8].

To date, most investigations related to *Lycopodii Herba* (*L. Herba*) and its components have focused on its isolation [6,9,10], identification [5], synthesis [11], and biological activity evaluation [9,12,13]. It has been revealed that *Lycopodium* alkaloids, which possess unprecedented diverse heterocyclic structures, are the major constituents of *L. Herba*. This herb has also been found to contain triterpenoids [14,15], anthraquinones [16], volatile oils [17], and flavonoids [18]. According to the chemist Ayer [19], *Lycopodium* alkaloids can be divided into four structural types; namelylycopodine-type, lycodine-type, fawcettimine-type, and miscellaneous-type, which typically contain lycodoline (LDL), α-obscurine (OSR), fawcettimine, and phlegmarine [13], respectively.

*Lycopodium* alkaloids have been claimed to have potent bioactivities, such as anti-inflammation, antitumor, and cholineasterase inhibitory activity [20,21,22], among which huperzine A has been approved for use as a drug for the treatment of Alzheimer’s disease (AD). These inspire researchers to focus on diverse *Lycopodium* alkaloids and *Lycopodium* genus plants. Our preliminary study has revealed that *L. Herba* could ameliorate learning and memory deficits in the AD mouse model, in which *Lycopodium* alkaloids may play vital roles in brain disorders. Therefore, studies on in vivo characteristics of the major *Lycopodium* alkaloids from *L. Herba*, especially characteristics of brain tissue distribution, are helpful to better understand the action mechanism and therapeutic efficacy of this plant. Until now, there has been only one report on the pharmacokinetics of OSR in rats after intragastrical (i.g.) adminidatration of ethanol extract of *L. Herba* [23]. However, the HPLC-UV method used in that study suffered from the disadvantages of tedious sample pretreatment procedures, long analysis time, low selectivity, and sensitivity. Furthermore, the therapeutic effects of traditional Chinese medicines usually depend on the synergy of multiple constituents, which suggests that the analysis of only one component is insufficient [24]. Hence, developing a simple and sensitive method for the simultaneous quantification of multiple *Lycopodium* alkaloids in a biological matrix would be beneficial for pharmacokinetic and tissue distribution studies of the alkaloids after i.g. administration of *L. Herba* extract (LHE).

The aim of the present study is to establish and validate a fast and sensitive LC-MS/MS method for simultaneous analysis of LDL, OSR, and *N*-demethyl-α-obscurine (DOR) in rat plasma and brain tissue. The method was fully validated in terms of selectivity, linearity, precision, accuracy, extraction recovery, and stability, and then applied to pharmacokinetic and brain tissue distribution studies of the alkaloids in rats. To the best of our knowledge, this is the first report to detail the determination of multiple *Lycopodium* alkaloids in rat biological matrices using an LC-MS/MS method, and the first investigation of the in vivo characteristics of the alkaloids in rats after i.g. administration of LHE.

## 2. Results and Discussion

### 2.1. Optimization of LC-MS/MS Conditions

**Figure 1:**
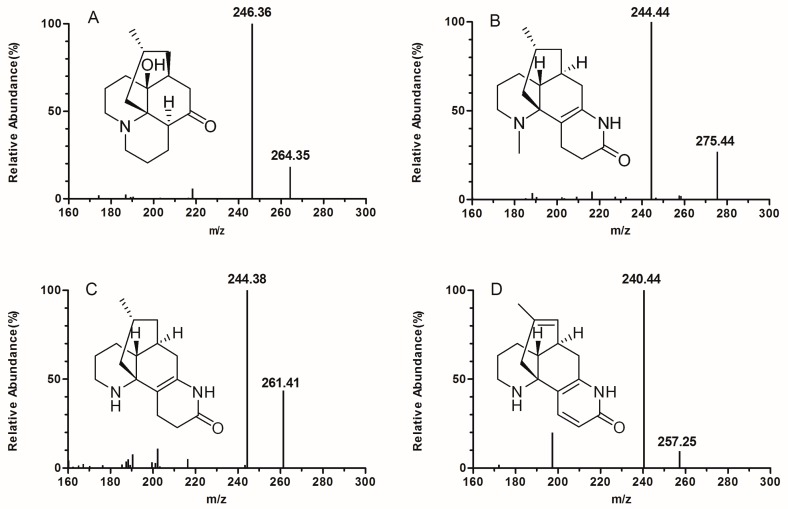
Full-scan product ion spectra of lycodoline (LDL) (**A**), α-obscurine (OSR) (**B**), *N*-demethyl-α-obscurine (DOR) (**C**), and Huperzine B (HZB) (**D**); internal standard, (IS).

Huperzine B (HZB) was chosen as the internal standard (IS) given its similar chemical structure to the analytes. Due to the alkaloids possess amino groups, we used selective reaction monitoring (SRM) in positive electrospray ionization mode. Different MS/MS parameters were optimized to obtain better selectivity and higher MS responses, especially the collision energy (CE). For example, the precursor ion used for LDL was [M + H]^+^ at *m/z* 264 Da, and the product ion was *m/z* 246 Da at 27 eV. The full-scan product ion spectra of the analytes and IS are presented in Figure 1. Their ion transitions and CE are summarized in Table 1.

Different chromatographic conditions were tested in order to obtain good peak shapes, adequate retentions, and high responses for the analytes with few differences in polarity exhibited. Methanol/water and acetonitrile/water mixtures were tested for the mobile phase, and methanol/water mixture was found to provide higher MS responses and better peak shapes. Moreover, different additives were investigated, including 0.01%, 0.05%, and 0.1% formic acid and 5 mM ammonium acetate. A quantity of 0.05% formic acid spiked to the mobile phase was able to improve peak shapes and increase MS responses of the analytes. As a result, 0.05% formic acid in water and methanol were used in the present study. After optimizing the gradient elution program, satisfactory chromatographic and mass spectrometric patterns were achieved for all the analytes.

### 2.2. Optimization of Sample Pretreatment

Stable extraction recovery, lower limit of quantification, and ignorable matrix effect are the standards when optimizing sample pretreatment procedures. The analyte response at the lower limit of quantification (LLOQ) should be at least five times higher than that of the blank samples. In this study, protein precipitation (PPT) approaches using methanol and acetonitrile were compared with liquid–liquid extraction (LLE) approaches using ethyl acetate, diethyl ether, and methyl tert-butyl ether. The results showed that the extraction recoveries of the analytes by LLE ranged from 28% to 76% with the LLOQs more than 20 ng/mL, while PPT provided higher extraction recoveries and lower LLOQs than LLE. However, PPT with acetonitrile produced bad peak shapes. Finally, PPT with methanol was adopted to prepare the biological samples because of its superior simplicity, higher and consistent extraction recovery, and negligible matrix effect.

### 2.3. Method Validation

#### 2.3.1. Selectivity, Crosstalk, and Carryover Effect

The selectivity of the method was assessed by determining possible matrix interferences by comparing the chromatograms of blank biological samples with those of blank biological matrix spiked with the analytes at LLOQ levels and real biological samples from LHE-treated rats. The crosstalk among the SRM channels of the analytes was evaluated by separately injecting a high concentration of individual analyte. The carryover effect was tested by analyzing the residual peaks in blank samples injected after an upper limit of quantification (ULOQ) sample.

**Figure 2:**
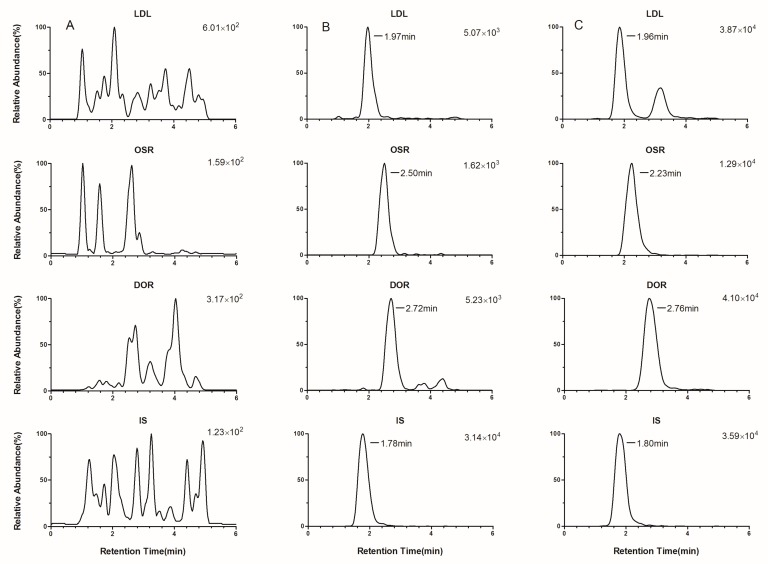
Typical chromatograms of the analytes and IS in rat plasma: blank plasma (**A**), blank plasma spiked with the alkaloids at the lower limit of quantification (LLOQ) (**B**), and plasma sample at 4 h after intragastrical (i.g.) administration of *L. Herba* extract (LHE) (**C**).

**Figure 3:**
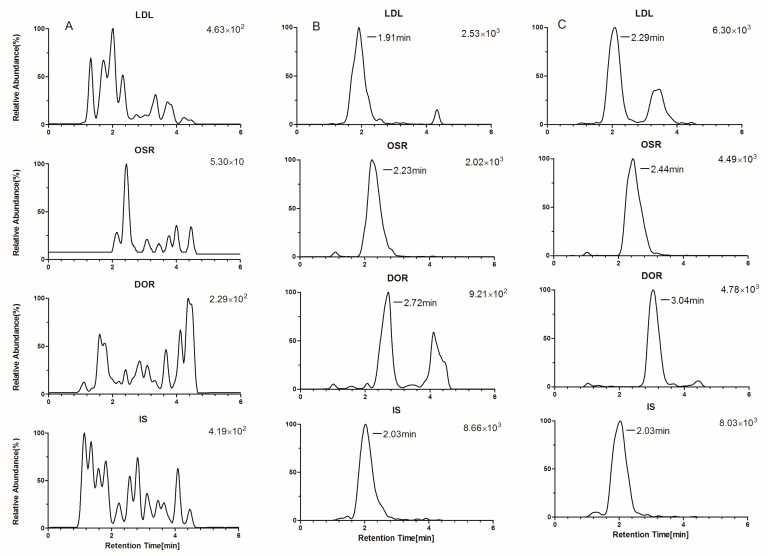
Typical chromatograms of the analytes and IS in rat brain tissue homogenate: blank brain tissue homogenate (**A**), blank brain tissue homogenate spiked with the alkaloids at LLOQ (**B**), and brain tissue homogenate sample at 3 h after after i.g. administration of LHE (**C**).

Typical chromatograms are presented in Figure 2 and Figure 3. From these, we can see that the responses of endogenous peaks at the retention times of the analytes were inside the validity limits, indicating the excellent selectivity of the method. No interfering and residual peaks were observed in the crosstalk and carryover effect items, indicating that the crosstalk and carryover effect were negligible.

#### 2.3.2. Linearity, LLOQ, and ULOQ

The linearity of the method was tested by analyzing the duplicate calibration standards on three consecutive days. The calibration curves were plotted as the peak area ratio of each analyte to IS versus the nominal concentration and then fitted to least-squares linear regression using 1/*x*^2^ as a weighing factor. The precision (the relative standard deviation, RSD) and accuracy (the relative error, RE) of LLOQ and ULOQ were determined by analyzing biological samples in six replicates at LLOQ and ULOQ levels, respectively.

The typical calibration curves, correlation coefficients (*r*), and linear ranges of the three analytes are presented in Table 2. The calibration curves showed good linearity (*r* ≥ 0.9911) over the test ranges. The LLOQs and ULOQs of the method were sufficient to be applied to in vivo studies, with acceptable precision (RSD ≤ 13.2% in plasma and ≤14.9% in brain at LLOQ; RSD ≤ 5.7% in plasma and ≤5.9% in brain at ULOQ) and accuracy (RE within −11.1% to 10.2% in plasma and −2.2% to 14.4% in brain at LLOQ; RE within −5.5% to −3.3% in plasma and 0.8% to 2.6% in brain at ULOQ).

#### 2.3.3. Precision and Accuracy

The precision and accuracy of the method were tested by analyzing quality control (QC) samples in six replicates over three consecutive days. The intra-day precision (RSD) and accuracy (RE) were tested by selecting the maximal deviation of theorical concentrations on one of the three days. The inter-day precision (RSD) and accuracy (RE) were tested by analyzing concentrations of all QC samples among three consecutive days. Table 3 summarizes the results. The intra-day and inter-day precision (RSD) of the three analytes did not exceed 14.2%, and the accuracy (RE) was from −9.6% to 9.6%. The results indicated that the present method is acceptable and reproducible for the determination of three alkaloids in rat biological samples.

#### 2.3.4. Extraction Recovery and Matrix Effect

The extraction recovery was determined by comparing the peak areas of each analyte spiked before and after the extraction procedure. The matrix effect was evaluated by comparing the peak areas of each analyte spiked after extraction with those in solutions containing equivalent contents. The results are listed in Table 4. The mean recovery of three alkaloids ranged from 81.2% to 110.1% (RSD ≤ 13.2). The IS recovery was 98.6% and 99.3% in the plasma and brain, respectively. The mean matrix effect ranged from 85.1% to 114.4% (RSD ≤ 13.5). The matrix effect of IS was 105.3% and 87.7% in the plasma and brain, respectively. All the results indicated stable extraction recovery and negligible endogenous interference and are thus suitable for determining the alkaloids in the rat biological matrix.

#### 2.3.5. Stability

The stability of the three analytes in rat biological matrix was tested by analyzing QC samples handled under different conditions at low and high QC levels, including short-term stability (storage for 24 h at room temperature), post-pretreatment stability (storage for 24 h after sample pretreatment), freeze–thaw stability (three cycles), and long-term stability (storage for 4 weeks at −80 °C). The results of this assessment are summarized in Table 5. The solution stability was assessed by comparing whether there was any degradation in the solutions after storage for 24 h at room temperature and four weeks at 4 °C. The results of this assessment are summarized in the Supplementary Materials. No obvious degradation of the analytes occurred under the above conditions, indicating that the analytes were stable in the rat biological matrix and solutions under the tested conditions.

### 2.4. Pharmacokinetic Study

**Figure 4:**
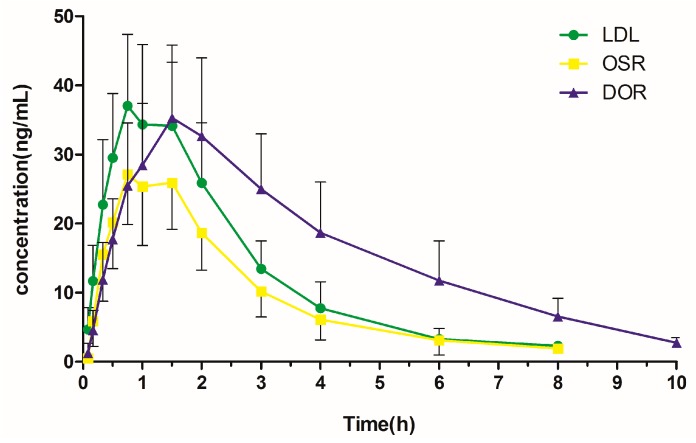
Mean plasma concentration–time curves of three alkaloids after i.g. administration of LHE (mean ± SD, *n* = 6).

We successfully applied the present method to the plasma pharmacokinetic study of three analytes in LHE-treated rats. The mean plasma concentration–time curves are presented in Figure 4. The pharmacokinetic parameters of the analytes are listed in Table 6, including the elimination half-life (*t*_1/2_), the maximum concentration (*C*_max_), the time to reach the maximum concentration (*T*_max_), the area under the concentration–time curve (AUC_0__→t_ and AUC_0__→∞_), and the volume of distribution (*V*_z_).

As shown in Table 6, the *T*_max_ values of LDL, OSR, and DOR ranged from 0.79 to 1.58 h, indicating that the alkaloids could be rapidly absorbed into the blood. The *C_max_* and AUC_0__→t_ values of the alkaloids were 27.70–37.25 ng/mL and 71.88–156.91 ng·h/mL, respectively. Although OSR was the most abundant component in LHE, its *C_max_* and AUC_0__→t_ values were the lowest among the three alkaloids. On the other hand, DOR had the lowest content in LHE, whereas it had the highest AUC_0__→t_ and AUC_0__→∞_ values. DOR plasma concentration declined with a *t*_1/2_ value of 2.24 h, whereas OSR were eliminated more rapidly with a *t*_1/2_ value of 1.37 h. The higher plasma exposure and slower elimination of DOR may be the result of the transformation from OSR after administration, as DOR is an *N*-desmethyl metabolite of OSR. The *C_max_* and *t*_1/2_ values OSR differ greatly from those reported in the literature [23]. This difference may reflect the use of different sources of *L. Herba*, different administrated doses of the alkaloids, and different analytical methods.

The maximum *V*_z_ value and the minimal plasma concentration found for OSR indicated that OSR might be distributed widely in rats. Many factors can affect drug distribution, such as cell membrane permeability, lipid–water partition coefficient, and the pH value of the fluid. Further experiments on the mechanisms of elimination and tissue distribution of the alkaloids are recommended.

### 2.5. Brain Tissue Distribution Study

**Figure 5:**
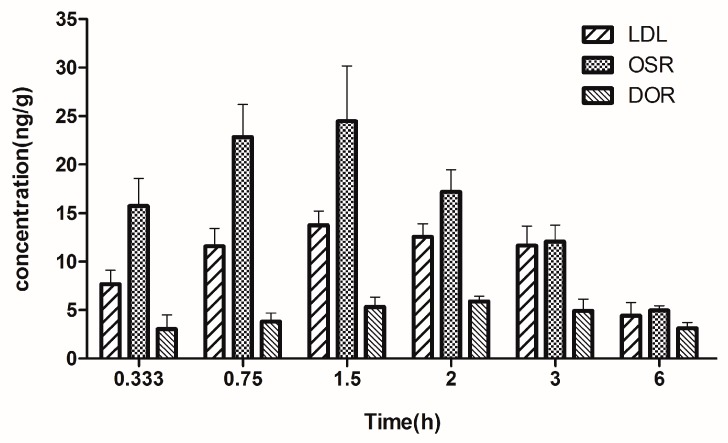
Mean brain tissue distribution histogram of three alkaloids in LHE-treated rats (mean ± SD, *n* = 6).

We also successfully used the present method for the determination of the three alkaloids in the brain tissue from LHE-treated rats. The brain tissue distribution histogram of the alkaloids is shown in Figure 5.

In the present study, the three alkaloids were detected in the rat brain samples at 0.333 h after drug administration. The maximal concentrations of LDL and OSR were 13.73 ± 1.34 and 24.46 ± 5.19 ng/g, respectively, which were observed at 1.5 h. In contrast, the highest DOR concentration of 5.89 ± 0.50 ng/g occurred at 2 h. The results showed that the three analytes could be distributed quickly in the rat brain tissues after i.g. administration of LHE. In the pharmacokinetic assessment, the concentrations of OSR were found to be the lowest among the three alkaloids in rat plasma, followed by LDL and DOR. In contrast, OSR was most extensively distributed in the brain, followed by LDL and DOR. These findings suggest that OSR passes through the BBB more easily and might play vital roles in the potential treatment of brain disorders with *L. Herba*. With higher polarity than OSR, DOR may have difficulty in transporting across the BBB. These findings are consistent with the results of *V*_z_ from the pharmacokinetic studies. At the last acquisition time point, the concentrations of LDL and OSR in some samples were below the LLOQ, whereas DOR was detectable in every brain sample. It is suggested that further studies should focus on the exploration of the characteristics of the alkaloids crossing the BBB. Furthermore, biological activities of the alkaloids—in particular, those related to brain disorders—are required to investigate using the present method as a reference [25].

## 3. Materials and Methods

### 3.1. Chemicals and Reagents

Analytical standard LDL was purchased from ChemFaces Biochemical Co. Ltd. (Wuhan, China). The OSR, DOR, and HZB (IS) standards were obtained from Guandao Biological Engineering Co. Ltd., ChemBest Research Laboratories Limited, and Yuanye Bio-Technology Co. Ltd. (Shanghai, China), respectively. All of the above-mentioned substances were of 98% purity. HPLC-grade formic acid and methanol were provided by Kermel Chemical Reagent factory and Concord Tech. (Tianjin, China), respectively. Water was supplied by the Wahaha Corporation (Hangzhou, China).

*L. Herba* was obtained from GuoDa Pharmacy (Shenyang, China), and authenticated by Prof. Jiuzhi Yuan from Shenyang Pharmaceutical University (Shenyang, China).

### 3.2. Preparation of *L. Herba* Extract

After grinding the dried plant material, 50 g powder of *L. Herba* was weighed accurately and extracted twice with 1 L 60% methanol solution by ultrasound-assisted extraction (UAE) for 50 min at room temperature. Then, the extracts were pooled and filtered. The combined filtrate was concentrated using a rotary evaporator, and then vacuum-dried to dryness. The LHE was obtained with a yield of 10.4% (*w*/*w*, extract/herb), and its LDL, OSR, and DOR contents were 793.8 μg/g, 999.8 μg/g, and 611.7 μg/g, respectively. The LHE was weighed accurately and dissolved in 0.05% carboxymethylcellulose sodium (CMC−Na) solution to acquire a concentration of 0.1 g/mL prior to the animal experiments.

### 3.3. Instrumentation for LC-MS/MS

Chromatographic separation was performed on a Shimadzu HPLC system (Kyoto, Japan) using a Thermo Syncronis-C_18_ column (50 mm × 2.1 mm, 5 μm) at 20 °C. The mobile phase of 0.05% formic acid (A) and methanol (B) was formed via gradient elution. The program was performed as follows: 0–0.5 min, 80% A; 1.0–5.0 min, 70% A; 5.5–10.0 min, 80% A. The flow rate was kept at 0.2 mL/min. The sample injection volume was 5 μL.

MS/MS analyses were performed using a Thermo TSQ Quantum Ultra triple-quadrupole mass spectrometer equipped with an electrospray ionization source (San Jose, CA, USA). The analytes were detected in positive SRM mode with an electrospray voltage of 4.2 kV and a capillary temperature of 370 °C. Nitrogen was used as the sheath gas (30 Arb) as well as the auxiliary gas (10 Arb), and argon was used as the collision gas (1.0 m Torr). LCquan quantitation software (version 2.5.6, ThermoFisher Scientific, Waltham, MA, USA) was used for data acquisition and analysis.

### 3.4. Preparation of Calibration Standards and QC Samples

Stock solutions of three analytes (0.2 mg/mL) and IS (0.2 mg/mL) were separately dissolved in methanol. Standard working solutions were prepared by successively diluting and mixing the stock solutions with a 50% methanol–water solution (2.50–200 ng/mL for LDL, 3.75–150 ng/mL for OSR, and 1.25–200 ng/mL for DOR). Similarly, the IS stock solution was diluted to a 150 ng/mL working solution for the pharmacokinetic study and a 60 ng/mL working solution for the brain tissue distribution study. All solutions were immediately stored at 4 °C.

Calibration standards were prepared via spiking blank plasma and brain tissue homogenate with the working solution mixture. The final concentrations in plasma were 2.0, 4.0, 10, 20, 40, and 80 ng/mL for LDL; 1.5, 3.0, 7.5, 15, 30, and 60 ng/mL for OSR; and 2.0, 4.0, 10, 20, 40, and 80 ng/mL for DOR. Similarly, the final concentrations in the brain tissue homogenate were 1.0, 1.6, 2.4, 4.0, 6.0, and 10 ng/mL for LDL; 2.0, 3.2, 4.8, 8.0, 12, and 20 ng/mL for OSR; and 0.5, 0.8, 1.2, 2.0, 3.0, and 5.0 ng/mL for DOR. The QC samples were prepared similarly at three concentration levels: 6.0, 24, and 60 ng/mL in plasma and 2.0, 5.0, and 8.0 ng/mL in brain tissue homogenate for LDL; 4.5, 18, and 45 ng/mL in plasma and 4.0, 10, and 16 ng/mL in brain tissue homogenate for OSR; and 6.0, 24, and 60 ng/mL in plasma and 1.0, 2.5, and 4.0 ng/mL in brain tissue homogenate for DOR.

### 3.5. Animals Experiments

Forty-two male Sprague-Dawley (SD) rats (weight 200 ± 20 g) were supplied by the Laboratory Animal Centre of Shenyang Pharmaceutical University (SYXK2014-0004, Shenyang, China). The experiments were approved by the Animal Ethics Committee of Shenyang Pharmaceutical University (SYPU-IACUC-C2017-11-20-203) and conducted in accordance with the principles for the Care and Use of Laboratory Animals [26]. Rats were housed in a constant temperature environment with 12 h/12 h light–dark cycles. Food and water were provided ad libitum.

All rats were randomly divided into seven groups (*n* = 6 per group); one for the pharmacokinetic study and others for the brain tissue distribution study. They were fasted for 12 h before being administered LHE i.g., but were allowed free access to water. The dosage of *L. Herba* was set at about 4.8 g/kg—close to 5 g/kg [23]. Blood samples (200 μL for each time point) were collected from the retinal venous plexus at 0, 0.083, 0.167, 0.333, 0.5, 0.75, 1.0, 1.5, 2.0, 3.0, 4.0, 6.0, 8.0, and 10.0 h after dosing into heparinized tubes. After centrifugation at 12,000 rpm for 5 min, the plasma was separated immediately and stored at −80 °C. At six different time points (0.333, 0.75, 1.5, 2.0, 3.0, and 6.0 h) following administration, the animals were anesthetized and exsanguinated, and the brain tissues were collected. Brain tissue samples were rinsed with normal saline solution, before being blotted, weighed, and homogenized with two volumes (*w*/*v*) of normal saline solution. After centrifugation at 12,000 rpm for 5 min, the supernatant was immediately separated and stored at −80 °C.

### 3.6. Samples Preparation

Prior to preparation, all frozen plasma and brain tissue homogenate samples were thawed at room temperature. PPT was used to extract the analytes from biological samples. Each 50 μL plasma sample was spiked with 20 μL of IS working solution (150 ng/mL), while 20 μL of IS working solution (60 ng/mL) was added to each 50 μL brain tissue homogenate sample. The mixtures were vortexed for 30 s and then 150 μL methanol was added for protein precipitation. The mixtures were vortexed again for 5 min, followed by 5 min centrifugation at 12,000 rpm. Finally, 5 μL of the supernatant was used for LC-MS/MS analysis.

### 3.7. Method Validations

Validation of the above method was in accordance with the current U.S. Food and Drug Administration (FDA) Guidance for Industry on Bioanalytical Method Validation [27].

### 3.8. Plasma Pharmacokinetic Study

The measured plasma concentrations of the analytes in the LHE-treated rats were analyzed using DAS software (version 2.0, Chinese Pharmacological Society, Beijing, China). The pharmacokinetic parameters of the three analytes were determined by non-compartmental analysis. The mean plasma concentration–time curves were presented using Graphpad Prism (version 5.0, Graghpad Software, San Diego, CA, USA).

### 3.9. Brain Tissue Distribution Study

The measured brain concentrations of the analytes were analyzed using Excel. The factual concentrations of the analytes in rat brain tissues were finally expressed in ng/g by the following equation: Cf = Cm × Vm/W, where Cf, Cm, Vm, and W represent the factual concentration (ng/g), the measured concentration (ng/mL), the homogenate volume (mL), and the weight (g) of the brain, respectively. The brain tissue distribution histograms were presented using Graphpad Prism (version 5.0).

## 4. Conclusions

This study developed and validated a fast and sensitive LC-MS/MS method for the simultaneous determination of three alkaloids from *L. Herba* in rat plasma and brain tissue. The method was successfully applied to study the plasma pharmacokinetics and brain tissue distribution in LHE-treated rats. The alkaloids were shown to be rapidly absorbed into the blood, and then also eliminated rapidly. All could go through the BBB into the brain and may thus play potential roles in the treatment of brain disorders with *L. Herba*. On the basis of the literature review, this is the first study to simultaneously determine multiple alkaloids from *L. Herba* in rat biological samples. Furthermore, it is the first application of the current method to plasma pharmacokinetic and brain tissue distribution studies of the alkaloids in LHE-treated rats. The established method can be used as a reference for any laboratory given its simple and reproducible extraction procedure and analytical conditions, and it provides a new research approach to expand preclinical studies of *L. Herba*.

## Figures and Tables

**Table 1:** MS/MS parameters of three alkaloids and internal standard (IS) in positive electrospray ionization (ESI^+^) mode. LDL—lycodoline; OSR—α-obscurine; DOR—*N*-demethyl-α-obscurine; CE—collision energy.

Analytes	Precursor *m*/*z* [M + H]^+^	Product *m*/*z*	CE (eV)
LDL	264.0	246.0	27
OSR	275.0	244.0	24
DOR	261.0	244.0	21
IS	257.0	240.0	20

**Table 2:** The linearity, lower limit of quantification (LLOQ), and upper limit of quantification (ULOQ) for the determination of three alkaloids in rat biological samples.

Biosamples	Analytes	Regression Equation	*r*	Linear Range (ng/mL)	LLOQ (ng/mL)	ULOQ (ng/mL)
plasma	LDL	y = 0.07541 x + 0.00113	0.9984	2.0–80	2.0	80
	OSR	y = 0.03665 x − 0.00666	0.9946	1.5–60	1.5	60
	DOR	y = 0.05349 x + 0.00171	0.9959	2.0–80	2.0	80
brain	LDL	y = 0.22353 x + 0.06646	0.9911	1.0–10	1.0	10
	OSR	y = 0.11798 x − 0.01198	0.9936	2.0–20	2.0	20
	DOR	y = 0.17938 x − 0.01427	0.9916	0.5–5.0	0.5	5.0

**Table 3:** Precision and accuracy for the determination of three alkaloids in rat biological samples (*n* = 6). RE—relative error; RSD—relative standard deviation.

Biosamples	Analytes	Theorical Concentration (ng/mL)	Intra-Day			Inter-Day		
			Measured Concentration (ng/mL)	RE (%)	RSD (%)	Measured Concentration (ng/mL)	RE (%)	RSD (%)
plasma	LDL	6.0	5.73	−4.5	7.5	5.99	−0.2	7.4
		24	25.25	5.2	6.5	24.34	1.4	6.3
		60	63.76	6.3	7.3	59.94	−0.1	7.1
	OSR	4.5	4.77	6.1	12.3	4.68	3.9	9.6
		18	17.12	−4.9	5.2	17.91	−0.5	5.4
		45	43.06	−4.3	7.0	43.90	−2.4	6.5
	DOR	6.0	6.38	6.3	9.6	6.25	4.2	7.8
		24	26.22	9.2	8.7	25.24	5.2	7.6
		60	65.76	9.6	12.3	63.12	5.2	8.7
brain	LDL	2.0	1.82	−8.8	14.2	1.94	−3.0	13.2
		5.0	4.60	−8.1	13.4	4.75	−4.9	10.1
		8.0	7.37	−7.8	9.0	7.76	−3.0	7.8
	OSR	4.0	3.61	−9.6	11.4	3.94	−1.5	12.1
		10	10.80	8.0	12.0	10.01	0.1	10.9
		16	17.37	8.6	10.5	16.31	1.9	10.1
	DOR	1.0	0.95	−4.7	13.6	0.98	−2.2	11.3
		2.5	2.37	−5.3	13.3	2.45	−2.0	9.9
		4.0	3.70	−7.5	11.3	3.78	−5.4	9.6

**Table 4:** Matrix effect and extraction recovery for the three alkaloids and the IS in the rat biological samples (*n* = 6).

Biosamples	Analytes	Concentration	Matrix Effect		Extraction Recovery	
		(ng/mL)	Mean (%)	RSD (%)	Mean (%)	RSD (%)
plasma	LDL	6.0	109.5	8.4	83.5	5.1
		24	114.4	4.3	104.8	8.0
		60	95.1	4.2	96.3	5.5
	OSR	4.5	100.2	13.5	102.5	8.2
		18	100.7	7.4	110.1	10.1
		45	97.3	2.1	105.2	6.6
	DOR	6.0	88.8	7.6	103.7	3.3
		24	103.5	6.8	98.7	8.4
		60	97.2	4.1	104.6	6.0
	IS	150	105.3	9.1	98.6	7.6
brain	LDL	2.0	91.9	9.4	81.5	5.8
		5.0	92.1	9.1	91.9	8.1
		8.0	91.2	3.1	85.5	3.3
	OSR	4.0	86.3	7.1	84.2	10.9
		10	88.8	6.3	91.1	5.2
		16	85.1	6.2	94.9	6.7
	DOR	1.0	86.0	2.9	81.2	13.1
		2.5	94.8	10.6	83.7	13.2
		4.0	85.5	6.3	82.7	6.0
	IS	60	87.7	8.7	99.3	10.4

**Table 5:** Stability of three alkaloids under various conditions (*n* = 3).

Samples	Analytes	Concentration (ng/mL)	Stability							
			Short-Term		Post-Preparation		Three Freeze–Thaw		Long-Term	
			RE%	RSD%	RE%	RSD%	RE%	RSD%	RE%	RSD%
plasma	LDL	6.0	3.9	1.0	6.3	8.7	2.7	4.7	9.0	2.6
		60	−4.4	8.4	1.6	5.3	1.8	4.5	9.2	6.7
	OSR	4.5	10.0	12.0	7.7	8.8	7.1	13.4	8.2	4.3
		45	−4.7	7.5	3.6	7.1	−2.9	5.1	6.6	5.5
	DOR	6.0	8.6	12.3	11.8	11.8	10.9	12.7	12.5	6.3
		60	3.5	6.9	4.9	8.5	5.5	3.0	11.6	4.9
brain	LDL	2.0	−9.1	9.3	−2.5	13.6	3.0	9.7	2.1	12.6
		8.0	−5.0	13.5	2.5	9.4	3.4	8.8	−5.7	9.3
	OSR	4.0	−4.0	12.5	−8.6	14.3	−2.4	11.0	−6.8	9.1
		16	−5.1	11.0	−3.0	14.8	2.0	8.7	−4.4	10.0
	DOR	1.0	−4.9	10.4	−9.0	10.4	−3.2	11.2	−2.0	12.5
		4.0	−3.5	9.4	5.1	9.2	3.2	13.3	−4.0	9.4

**Table 6:** Pharmacokinetic parameters of three alkaloids in *L. Herba* extract (LHE)-treated rats (mean ± SD, *n* = 6). AUC—area under the concentration–time curve.

Parameters	LDL	OSR	DOR
*C_max_* (ng/mL)	37.25 ± 10.60	27.70 ± 7.70	35.53 ± 10.44
*T_max_* (h)	0.79 ± 0.10	0.92 ± 0.30	1.58 ± 0.20
*t_1/2_* (h)	1.27 ± 0.31	1.37 ± 0.44	2.24 ± 0.25
AUC_0→t_ (ng·h/mL)	101.25 ± 33.96	71.88 ± 24.13	156.91 ± 55.38
AUC_0→∞_ (ng·h/mL)	104.92 ± 34.85	78.49 ± 28.22	167.11 ± 54.78
*V*_z_ (L/kg)	7.58 ± 3.38	13.30 ± 4.83	6.68 ± 2.95

## Supplementary Materials

The following are available online, Table S1: Stability of three alkaloids in solution under various conditions (*n* = 3), Figure S1: Typical chromatograms of three alkaloids and IS in *L. Herba* extract.
