# Supplementary material for: Pharmacokinetic Studies of Three Alkaloids in Rats After Intragastrical Administration of Lycopodii Herba Extract by LC-MS/MS
Source: Molecules. 2019 May 19;24(10):1930. doi: 10.3390/molecules24101930 (PMC6572600; doi:10.3390/molecules24101930)
Supplement: Supplementary file 1 [file molecules-24-01930-s001.pdf]

Pharmacokinetic Studies of Three Alkaloids in Rats After Intragastrical Administration of *Lycopodii Herba* Extract by LC–MS/MS

Dongke Ma, Xiaoting Gu, Xin Wang, Youping Liu and Xin Di *

1. Solution Stability

The solution stability was assessed by comparing whether there was any degradation in the solutions after storage for 24 h at room temperature and 4 weeks at 4 °C. The results of this assessment are summarized in Table S1.

Table S1. Stability of three alkaloids in solution under various conditions (n = 3).

Sample	Analytes	Concentration (ng/mL)	Stability			
			Short-Term		Long-Term	
			RE%	RSD%	RE%	RSD%
solution	LDL	2.0	–3.8	3.6	–3.1	7.6
		8.0	–4.4	3.7	–2.3	1.1
		60	3.0	2.1	–3.2	2.1
	OSR	4.0	5.3	3.2	3.9	2.2
		16	–4.6	2.6	–3.9	7.0
		45	5.6	2.9	4.7	3.1
	DOR	1.0	–4.8	6.8	4.9	5.8
		6.0	–5.4	7.0	–5.1	2.7
		60	4.4	3.5	2.3	4.0

2. Simultaneous determination of three alkaloids from *L. Herba* Extract by LC–MS/MS

Chromatographic separation was performed using the mobile phase of 0.05% formic acid (A) and methanol (B) bia gradient elution. The program was performed as follows: 0–0.8 min, 82% A; 1.0–8.5 min, 70% A; 9.0–14.0 min, 82% A. The flow rate was kept at 0.2 mL/min.

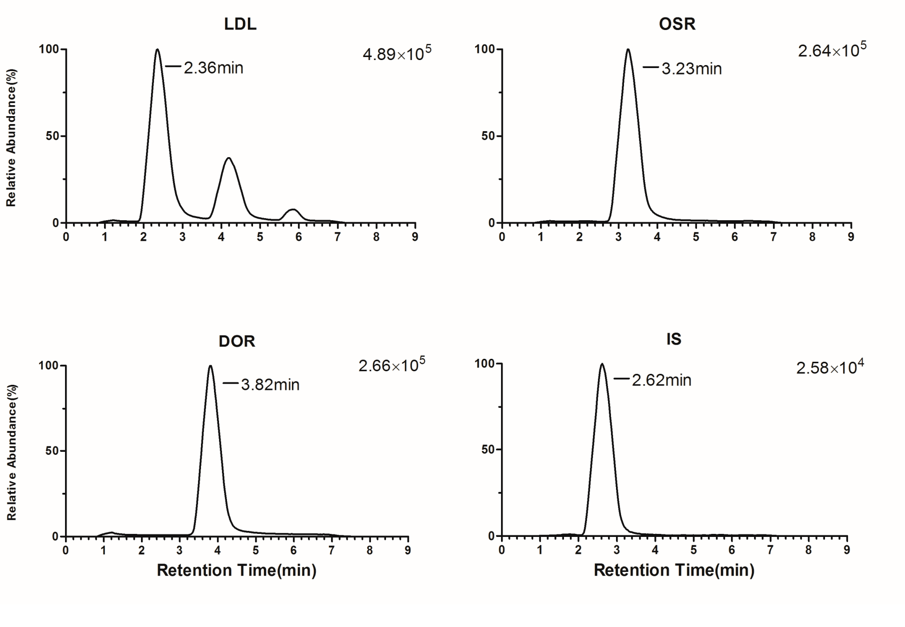

Figure S1. Typical chromatograms of three alkaloids and IS in *L. Herba* extract.
